# Supplementary material for: Identification of photoperiod-induced specific miRNAs in the adrenal glands of Sunite sheep (Ovis aries)
Source: Front Vet Sci. 2022 Jul 22;9:888207. doi: 10.3389/fvets.2022.888207 (PMC9354845; doi:10.3389/fvets.2022.888207)
Supplement: Supplementary file 1 [file Data_Sheet_1.ZIP › Supplementary file/miRDeep2 file.zip!/miRDeep2 file/Novel_Structure/SPLP42A2/Novel_203.pdf]

| | | |
|---|---|---|
| Provisional ID | : | NC_019465.2_18658 |
| Score total | : | 4.6 |
| Score for star read(s) | : | 3.9 |
| Score for read counts | : | 0.6 |
| Score for mfe | : | -1.5 |
| Score for randfold | : | 1.6 |
| Score for cons. seed | : | |
| Total read count | : | 13 |
| Mature read count | : | 12 |
| Loop read count | : | 0 |
| Star read count | : | 1 |

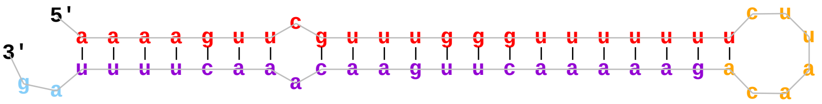


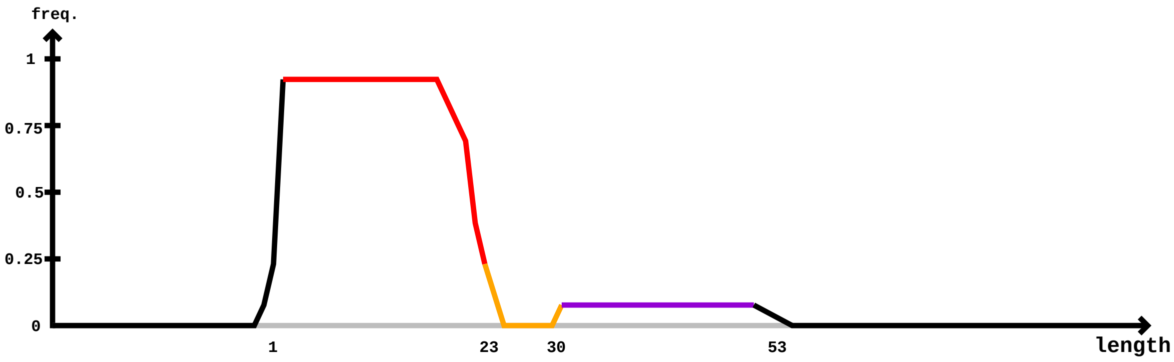


**Mature** **Star**

```
5'- uuacuuugcauugguuugaccgaaaaguucguuuggguuuuuuucuuaacagaaaaacuugaacaaacuuuuagcuaacccaauauuugucaccuuaugauagaaaggaaac   -3'    obs
    uuacuuugcauugguuugaccgaaaaguucguuuggguuuuuuucuuaacagaaaaacuugaacaaacuuuuagcuaacccaauauuugucaccuuaugauagaaaggaaac          exp
    .........(((((.(((.(..(((((((.((((((((((((((......)))))))))))))).))))))).).))).))))).(((((((.....)))))))........    reads      mm          sample
    ....................cgaaaaguucguuuggguuuuu......................................................................    1          0           seq
    .....................gaaaaguucguuuggguuuuu......................................................................    2          0           seq
    ......................aaaaguucguuuggguu.........................................................................    1          0           seq
    ......................aaaaguucguuuggguuu........................................................................    1          0           seq
    ......................aaaaguucguuuggguuuG.......................................................................    1          1           seq
    ......................aaaGguucguuuggguuuuu......................................................................    1          1           seq
    ......................aaaaguucguuuggguuuuuA.....................................................................    2          1           seq
    ......................aaaaguucguuuggguuuuuuu....................................................................    1          0           seq
    ......................aaaaguucguuuggguuuuuGu....................................................................    2          1           seq
    ...................................................gaaaaacCugaacaaacuuuu........................................    1          1           seq
```
